# Individual Actin Filaments in a Microfluidic Flow Reveal the Mechanism of ATP Hydrolysis and Give Insight Into the Properties of Profilin

**DOI:** 10.1371/journal.pbio.1001161

**Published:** 2011-09-27

**Authors:** Antoine Jégou, Thomas Niedermayer, József Orbán, Dominique Didry, Reinhard Lipowsky, Marie-France Carlier, Guillaume Romet-Lemonne

**Affiliations:** 1 Laboratoire d'Enzymologie et Biochimie Structurales, Centre de Recherche de Gif, CNRS, Gif-sur-Yvette, France; 2 Theory and Biosystems, Max Planck Institute of Colloids and Interfaces, Potsdam, Germany; Yale University, United States of America

## Abstract

The hydrolysis of ATP associated with actin and profilin-actin polymerization is pivotal in cell motility. It is at the origin of treadmilling of actin filaments and controls their dynamics and mechanical properties, as well as their interactions with regulatory proteins. The slow release of inorganic phosphate (Pi) that follows rapid cleavage of ATP gamma phosphate is linked to an increase in the rate of filament disassembly. The mechanism of Pi release in actin filaments has remained elusive for over 20 years. Here, we developed a microfluidic setup to accurately monitor the depolymerization of individual filaments and determine their local ADP-Pi content. We demonstrate that Pi release in the filament is not a vectorial but a random process with a half-time of 102 seconds, irrespective of whether the filament is assembled from actin or profilin-actin. Pi release from the depolymerizing barbed end is faster (half-time of 0.39 seconds) and further accelerated by profilin. Profilin accelerates the depolymerization of both ADP- and ADP-Pi-F-actin. Altogether, our data show that during elongation from profilin-actin, the dissociation of profilin from the growing barbed end is not coupled to Pi release or to ATP cleavage on the terminal subunit. These results emphasize the potential of microfluidics in elucidating actin regulation at the scale of individual filaments.

## Abstract 2

A novel microfluidic approach allows the analysis of the dynamics of individual actin filaments, revealing both their local ADP/ADP-Pi-actin composition and that Pi release is a random mechanism.

## Author Summary

Actin proteins assemble into microfilaments that control cell shape and movement by polymerizing or depolymerizing. These actin monomers can bind ATP or ADP molecules. The incorporation of an ATP-actin monomer into a growing filament results in rapid cleavage of ATP into ADP and inorganic phosphate (Pi), followed by a slower release of Pi. As a consequence, actin filaments are composed mainly of ADP- and ADP-Pi-actin subunits, which have different depolymerization kinetics and mechanical properties, and can be targeted specifically by regulatory proteins that affect filament function. Hence, the understanding of many cellular processes requires a knowledge of the ADP/ADP-Pi composition of actin filaments at a molecular scale. This has so far remained elusive because traditional studies rely on measuring an average over many filaments in solution. To address this issue, we developed a microfluidics setup to monitor individual filaments with light microscopy while rapidly changing their chemical environment. We find that depolymerization accelerates progressively and corresponds to an exponential ADP-Pi-actin profile in the filament, meaning that each subunit releases its Pi with the same rate. Our method also provides novel insight into the function of profilin, a protein important for regulation of actin dynamics in cells, thus demonstrating the method's potential in the functional analysis of actin regulators.

## Introduction

**Figure 1:**
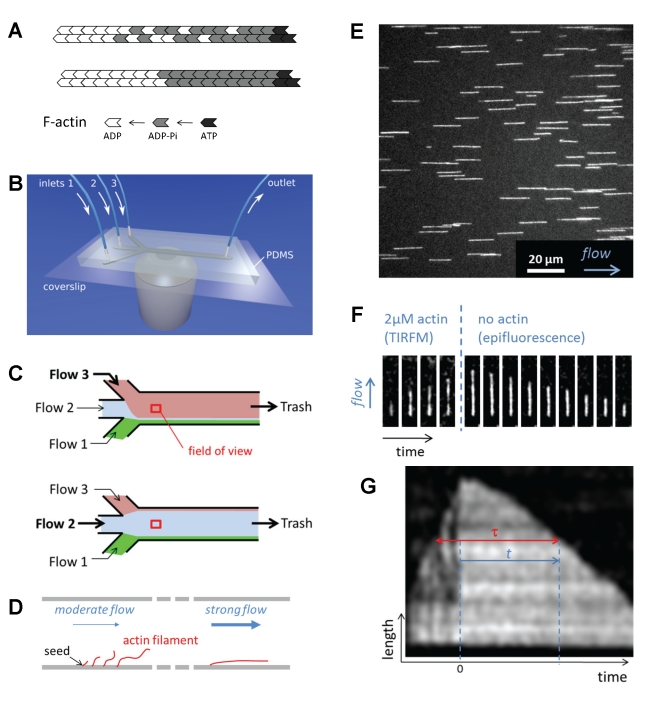
Microfluidics setup for the observation of individual actin filaments. (A) Random (top) or vectorial (bottom) model for Pi release in actin filments. (B) Microfluidics flow-cell on a microscope objective. (C) Flow-cell seen from above. The dominant laminar flow determines which medium occupies most of the flow-cell. (D) Side view of the flow-cell. When the microflow is sufficiently fast, the filaments align and remain close to the glass coverslip. (E) Epifluorescence image of filaments anchored to the coverslip of a flow-cell via spectrin-actin at their pointed end (left end), and aligned with the buffer flow (arrow). (F) Time lapse images of a single filament, observed in TIRF during elongation (1.5 µM actin, during 5 min) and epifluorescence during depolymerization. (G) Kymograph of the filament shown in (F). The age *τ* of the subunits that dissociate at time t of the depolymerization process is indicated by the red line.

Actin-based motile processes are driven by the polarized assembly of actin filaments [1]–[3]. As filaments elongate by endwise association of ATP-actin, ATP is rapidly cleaved into ADP-Pi. Slower release of Pi thus maintains a cap of ADP-Pi actin subunits at the growing filament barbed ends [4],[5]. The release of Pi destabilizes actin-actin bonds in the filament and lowers the rigidity of the polymer [6]. Although it is known that the rate of dissociation of ADP-Pi-actin from the barbed ends is about 10-fold slower than the rate of dissociation of ADP-actin [5],[7], the detailed molecular mechanism of Pi release from F-actin during growth of the filament has remained elusive [8],[9]. For Pi release, as for ATP γ-phosphate cleavage, two main mechanisms have been considered since long ago [5] and continue to receive attention, notably from theoretical studies [10]–[13]: in the random model, each ADP-Pi-actin subunit releases its Pi with equal probability, while the vectorial model assumes that Pi can only be released from an ADP-Pi-subunit adjacent to an ADP-subunit (Figure 1A). In the latter model, an ADP/ADP-Pi boundary propagates toward the growing barbed end that displays a strict ADP-Pi cap, while the cap has a mixed composition in the random model.

A detailed understanding of the mechanism of ATP hydrolysis on actin is required to further address how it may control or be affected by regulators of actin dynamics like profilin, capping proteins, or ADF/cofilin that bind differently to ADP- or ADP-Pi-actin. In particular, the role of ATP hydrolysis in the function of profilin has been a subject of debate since experimental evidence is available both against [14],[15] and in favor of [16] the coupling of ATP hydrolysis to filament elongation from profilin-actin. This point is difficult to address theoretically due to the many reactions that need to be considered [17],[18] and a full understanding of the effect of profilin on actin dynamics requires additional experimental data.

Bulk solution studies, which provide measurements averaged on large numbers of filaments, have failed to deliver conclusive answers on the molecular mechanism of ATP hydrolysis. Recent theoretical studies [10],[13],[19] have proposed to use the length fluctuations observed on individual actin filaments near the critical concentration [20],[21] as a way to discriminate between the different models for ATP hydrolysis, but the required measurements seem difficult to achieve experimentally. In the present work, individual filaments growing with an ADP-Pi cap are rapidly switched to depolymerizing conditions using an original setup based on microfluidics, inspired from DNA studies (Figure 1B–C) [22]. The off-rate of actin subunits at the barbed end depends on the bound nucleotide, and provides insight into the ADP/ADP-Pi composition of the filament, and the mechanism of Pi release.

## Results

### Monitoring Elongation and Disassembly of Individual Filaments

Spectrin-actin seeds were adsorbed on the surface of the glass coverslip (Figure 1D–E), at the bottom of the flow cell. Filaments grew from these seeds at a constant rate by flowing in a fluorescently labeled G-actin solution for a few minutes. Growth up to a few micrometers in length was monitored by Total Internal Reflection Fluorescence (TIRF) microscopy. In the field of view, depolymerization from the free barbed ends was then triggered by switching to the flow channel that contained no actin (Figure 1C,F–G and Video S1). The transition from elongation conditions to depolymerization conditions took place in less than a second. During depolymerization, epifluorescence microscopy could be used due to the absence of fluorescent background from solution.

This microfluidic technique avoids the drawbacks of standard individual filament observations: The actin filaments are anchored at their pointed ends to the surface by the spectrin-actin seeds, but not immobilized along their length by anchor proteins. Filaments are aligned by the flow, making the monitoring of their contour length and the derivation of kymographs straightforward and accurate. In the conditions of our experiments, no pointed end elongation of filaments from the spectrin-actin seeds was detected. We verified that in the used range, the fraction of labeled actin, exposure time, and flow rate did not affect the kinetic parameters at the barbed end (see Materials and Methods). Pauses in the depolymerization of individual filaments have been observed, reminiscent of the recently reported “filament stabilization” [23]. On a time scale of the order of 10 min, an increasing number of filaments exhibit pauses. The interruption times, at which these pauses begin, strongly vary from filament to filament. However, the depolymerization traces up to the interruption times are very similar, indicating that they are independent of the pauses (Figure S1). For more details on pauses, see Text S1 and Figure S2.

### Exponential Profile of ADP-Pi-Actin in Filaments

**Figure 2:**
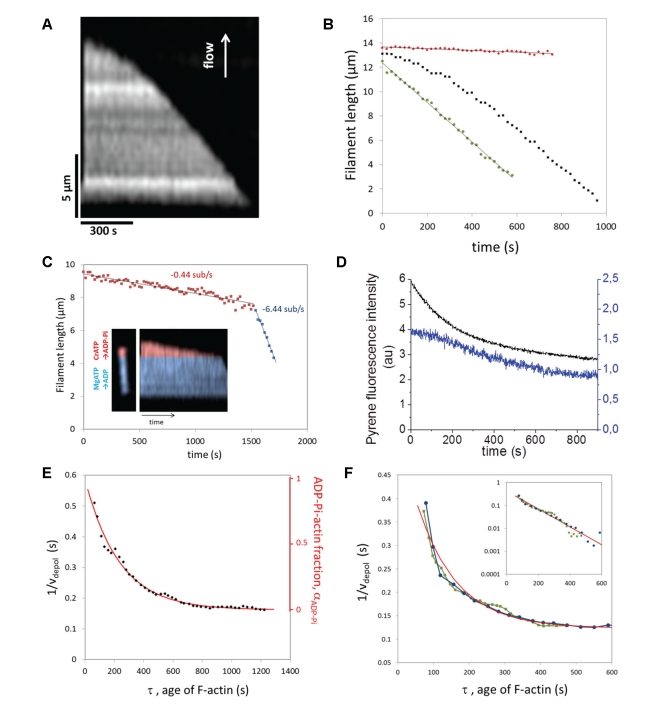
Depolymerization of actin filaments and measurement of their ADP-Pi content. (A) Kymograph of a depolymerizing actin filament, after elongation with 1.5 µM MgATP-actin for 5 min. (B) Depolymerization of the MgATP-actin filament from (A) (black), of an ADP-actin filament (green) and of a CrATP-actin filament (red). (C) Depolymerization of a filament constructed with an artificial strict ADP-Pi cap, mimicking vectorial Pi release. Inset: fluorescence image of the filament, showing the ADP-Pi and ADP regions in pseudo colors, and kymograph of its depolymerization. (D) Bulk solution depolymerization assays. Filaments are grown from 0.25 nM spectrin-actin seeds at 2 µM actin (50% pyrene labeled) and depolymerized by 6-fold dilution with 5 µM Latrunculin A, at times at which 20% (blue curve, right *y*-axis) or 75% (black curve, left *y*-axis) of actin has polymerized. Under these conditions, the barbed ends generated by spontaneous nucleation are negligible. (E) 1/*v_depol_* versus the age of F-actin for the filament shown in (A) and (B). This curve is fitted by an exponential (red line) with rate constant *k_r_* = 0.0043 s^−1^, directly representing Pi decay in the filament. (F) Pi decay profiles for filaments elongated with 2 µM actin during 2.5 (green) or 10 min (blue), compared with an exponential fit (red line). Inset: log-linear plot of 
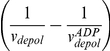
 for the same data.

Filaments grown from MgATP-actin depolymerize at a pace that accelerates progressively, on a time scale of a few minutes, as the nucleotide content of the depolymerizing region evolves from ADP-Pi-rich to ADP-rich (Figure 2A–B and Figure S1). We assume that ATP-F-actin is a short-lived species and cannot be detected in our measurement [4],[14]. Filaments grown from MgADP-actin depolymerize at a constant rate 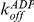 = 5.8±0.4 subunits/s (mean ± standard deviation, *N* = 11 filaments) (Figure 2B). Filaments assembled from MgATP-actin that were aged at a constant length in a flow of 0.1 µM G-actin (the barbed end critical concentration) subsequently depolymerized at this constant rate as well (Figure S3). ADP-Pi actin filaments also depolymerize at a constant rate: 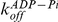 = 0.16±0.07 subunits/s (*N* = 13) was measured for filaments depolymerizing in the presence of a saturating concentration of 100 mM Pi, and 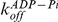 = 0.33±0.16 subunits/s (*N* = 21) was measured for filaments assembled from CrATP-actin, which cannot release its Pi [24]. These depolymerization rates confirm earlier measurements in bulk solution [5] as well as measurements on single actin filaments [25], while other single filament studies have reported much slower rates for ADP-actin [23] possibly because of filament-surface interactions.

To mimic the effect of vectorial Pi release, a strict ADP-Pi cap was artificially constructed by elongating filaments sequentially from MgATP-actin then CrATP-actin. These artificial filaments exhibited a sharp transition from a slow to a rapid rate of depolymerization (Figure 2C), differing from the smooth increase in depolymerization rate observed for filaments assembled only from MgATP-actin (Figure 2A–B).

Evidence for a slowly depolymerizing ADP-Pi cap was also observed in bulk solution measurements using pyrenyl-actin fluorescence. Filaments growing in a synchronous fashion from spectrin-actin seeds displayed an accelerating depolymerization when they were switched to depolymerizing conditions in early stages of assembly, while they displayed only the regular rapid depolymerization when switched to depolymerizing conditions upon approaching steady state (Figure 2D and Figure S4). These data corroborate the view [5] that in a regime of rapid barbed end growth, a large cap of “young” slowly dissociating ADP-Pi subunits exists, which vanishes when growth slows down due to depletion of the pool of actin monomers, upon approaching steady state of assembly. The rapid depolymerization of ADP-F-actin was followed by a decline in rate that may be due to the cumulated effect of pauses, which are observed in single filament experiments (Figure S1) but are not identifiable in the population of filaments in solution. Hence the kinetic analysis of depolymerization is feasible only on individual filaments and was carried out as follows.

The depolymerization velocity *v_depol_* of a segment of filament directly reflects its nucleotide composition. It can be estimated by local linear fits of the depolymerization curve (see Materials and Methods). Having monitored the elongation of the filaments, we know the age *τ* of the depolymerizing F-actin (Figure 1G and Materials and Methods) and we can hence determine *v_depol_*(τ). We assume that the dissociation rate of each actin subunit from the barbed end depends only on its nucleotide state, and not on that of its neighbors. In contrast, we make no hypothesis on Pi release, which takes place in the core of the filament and may be affected by the nucleotide state of neighboring subunits. Since Pi release is slow compared to barbed-end depolymerization, it does not take place significantly within a sufficiently small segment while this segment disassembles (see Text S1, equation 9). Thus, the depolymerization velocity *v_depol_* of such a segment is determined by its fractions *α_ADP–Pi_* of ADP-Pi- and (1−*α_ADP–Pi_*) of ADP-actin subunits, which implies
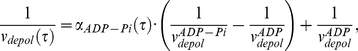
(1)where 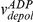 and 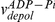 are the depolymerization velocities of ADP-actin and ADP-Pi-actin subunits, respectively. Thus, measuring 1/*v_depol_*(τ) for a depolymerizing filament provides a direct readout of *α_ADP–Pi_*(τ), the profile of Pi release in aging F-actin. Note that the age-dependent function *α_ADP–Pi_*(τ) can also be seen as the spatial profile of ADP-Pi-actin subunits along the filament, as age *τ* increases with the distance from the barbed-end. The function 1/*v_depol_*(τ) is shown in Figure 2E. The local protomer composition is well fitted by a single-exponential decay, *α_ADP–Pi_*(τ) = *e^−k_r_^^τ^*, showing that Pi release is a random process, with a rate constant *k_r_* = 0.0068±0.0021 s^−1^ (*N* = 20). This corresponds to a half-time of *ln*(2)/*k_r_* = 102 s. This value is comparable to previous measurements of the Pi release rate constant *k_r_* from bulk solution [4],[5],[26],[27] or individual filament [25] studies, with *k_r_* ranging from 0.002 to 0.006 s^−1^. Filaments elongated at different actin concentrations, i.e. different velocities, or for different durations all displayed the same age-dependence of depolymerization rate (Figure 2F), confirming that the ADP-Pi content depends only on the age of the F-actin, as expected for a random Pi release mechanism.

To validate this analysis of the experimental 1/*v_depol_*(τ) data, we have derived an exact formula for the observed filament length as a function of time (Text S1). Fitting the experimental data with this formula leads to the same conclusion as above: the best fit is obtained for random Pi release, with *k_r_* = 0.0074 s^−1^, 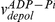 = 1.5 subunits/s, and 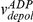 = 6.0 subunits/s. The age-dependent *α_ADP–Pi_*(*τ*) derived from equation (1) was further corroborated by stochastic simulations (Figures S5, S6, and S7).

### Phosphate Release Is Faster from the Barbed End

**Figure 3:**
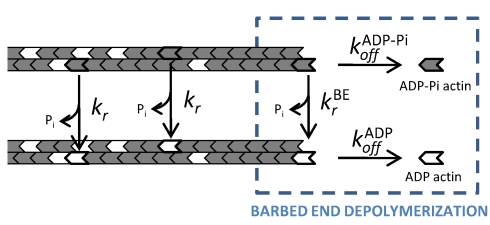
Phosphate release is enhanced at the barbed end. In the bulk of the filament, Pi is released randomly with rate constant *k_r_* = 0.0068±0.0021 s^−1^, regardless of the nucleotide state of the neighboring actin subunits. At the barbed end, Pi is released with rate constant 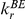 = 1.8±0.6 s^−1^. An ADP-Pi-actin subunit exposed at the barbed end during depolymerization can either dissociate as an ADP-Pi subunit, with rate 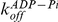, or release its Pi with rate 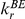 and then dissociate as an ADP-actin subunit, with rate 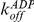. The resulting global rate constant for ADP-Pi-actin dissociation is referred to as 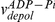. The presence of profilin does not affect *k_r_* but increases all the other rate constants.

Fitting 1/*v_depol_*(*τ*) using an exponential profile for *α_ADP–Pi_*(*τ*) in equation (1) also yielded values for 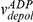 and 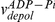. As the filament ages, its depolymerization velocity converges to 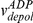 = 6.2±0.4 subunits/s (*N* = 20), in agreement with 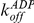 = 5.8±0.4 subunits/s (*N* = 11). At the onset of depolymerization, the filament depolymerizes with velocity 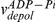 = 1.5±0.4 subunits/s (*N* = 20), which differs from 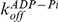  = 0.16±0.07 subunits (*N* = 13) since a terminal ADP-Pi-actin subunit can dissociate from the barbed end of the filament by two possible routes: (i) it dissociates as an ADP-Pi-actin subunit with a rate constant 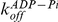 or (ii) releases its phosphate with a rate constant 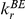 and subsequently dissociates as an ADP-actin subunit with a rate constant 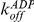 (Figure 3). The combination of these two routes leads to the depolymerization velocity 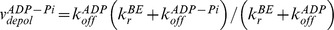, from which we can compute the Pi release rate 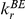 = 1.8±0.6 s^−1^ (half-time of 0.39 s) at the barbed end of the filament, which is orders of magnitude larger than the Pi release rate in the filament (see Text S1, equation 2). This confirms the enhanced Pi dynamics at the barbed end proposed by Fujiwara et al. [25] and our measurement corresponds to the lower limit of their estimated range for 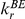. Depolymerizing ADP-actin filaments rapidly switch to a slow depolymerization rate when exposed to Pi (Figure S8), which indicates that, at least at the barbed end, Pi binds rapidly to F-actin and stabilizes its interactions with neighbors. Release of Pi in the core of the filament has been proposed to be kinetically limited by the slow conversion of the ATP hydrolysis transition state F-ADP-P* into F-ADP-Pi [28], which is supported by recent cryo-EM structural analysis of the ADP-Pi filament [29].

### Depolymerization Is Faster in the Presence of Profilin

**Figure 4:**
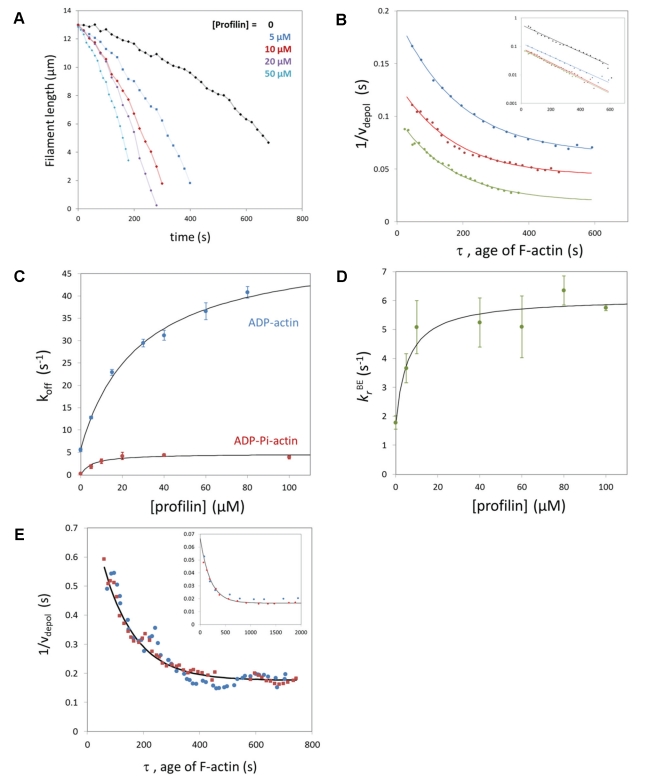
Effect of profilin on actin dynamics and phosphate release at the barbed end. (A)Time course of filaments depolymerizing in the presence of 0 (black), 5 µM (blue), 10 µM (red), 20 µM (purple), or 50 µM (cyan) profilin, after elongation with 1.5 µM actin for 5 min. (B) 1/*v_depol_* versus the age of F-actin measured on filaments depolymerizing with 5 µM (blue), 10 µM (red), and 80 µM (green) profilin, and exponential fits (lines). Inset: log-linear plots of 
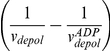
 for the same data, compared to a filament depolymerizing without profilin (black). (C) Effect of profilin on the off-rates of ADP-actin and ADP-Pi-actin (measured on CrATP-actin). The lines are saturation curves fitting the data, assuming that profilin is in rapid equilibrium with the barbed end of the filaments. (D) Effect of profilin on the Pi release rate constant at the barbed end, 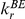, computed based on the molecular scheme shown in Figure 3, and fitted by a saturation curve assuming that profilin is in rapid equilibrium with the barbed end. (E) Pi decay profiles (1/*v_depol_* versus the age of F-actin) measured on filaments elongated with 2 µM actin and 6 µM (blue) or no (red) profilin, and depolymerized with no profilin, compared to an exponential profile with *k_r_* = 0.008 s^−1^ (line). Inset: Pi decay profiles (1/*v_depol_* versus the age of F-actin) measured on filaments elongated with 0.25 µM actin and 2 µM profilin (blue) or 0.2 µM actin and no profilin (red), and depolymerized with 100 µM profilin, compared to a profile computed with *k_r_* = 0.005 s^−1^ (line).

The still elusive issue of the effect of profilin on actin dynamics [17] can be addressed using our microfluidic setup. During depolymerization, the addition of profilin in the buffer accelerated the disassembly of actin filaments in a concentration-dependent manner (Figure 4A). This acceleration disappeared when switching to a flow channel containing no profilin (Figure S9) confirming that profilin is not binding to internal F-actin subunits. Consistently, the presence of profilin during depolymerization increases depolymerization velocities 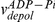 and 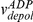 but does not affect the filament's ADP-Pi profile, which remains exponential with the same Pi release rate *k_r_* (Fig. 4B). We have monitored the depolymerization of MgADP-actin and CrATP-actin filaments at different profilin concentrations. We show that profilin increases 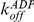, in agreement with earlier studies in solution [30], and that it also increases 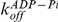 (Figure 4C). Having measured the impact of profilin on 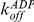, 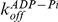, and 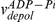, we can compute its impact on 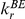 following the reaction scheme of Figure 3 and find that profilin accelerates Pi release at the barbed end of actin filaments (Figure 4D). Profilin has a higher affinity for ADP-Pi-actin than for ADP-actin at the barbed end, with dissociation constants of 5.9±2.4 µM and 28.1±5.1 µM, respectively, obtained by fitting the saturation curves (Figure 4C) assuming that profilin is in rapid equilibrium with the barbed end. These results are summarized in Table 1.

**Table 1:** Rate constants for the dissociation of actin monomers and inorganic phosphate (Pi) from filaments depolymerizing by their barbed ends.

	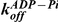 (s^−1^)	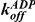 (s^−1^)	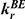 (s^−1^)	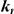 (s^−1^)
F-actin alone	0.16±0.07	5.8±0.4	1.8±0.6	0.0068±0.0021
with saturating [profilin]	4.7±0.4^a^	51.6±2.9^b^	6.1±0.3^a^	0.0065±0.0006^c^

a K_d_ = 5.9±2.4 µM for the binding of profilin to MgADP-Pi-actin at the barbed end.

b K_d_ = 28.1±5.1 µM for the binding of profilin to MgADP-actin at the barbed end.

c Measured at 100 µM profilin.

### Elongation from Profilin-Actin Results in the Same ADP-Pi-Actin Content

We have monitored filaments elongated with various amounts of G-actin (0.2 to 6 µM) and profilin (0 to 9 µM) and found that elongation from profilin-actin was 30% slower than from actin alone at the same concentration, as measured previously in solution [31]. Filaments elongated from profilin-MgATP-actin exhibit the same ADP-Pi profile when depolymerizing than filaments elongated from MgATP-actin (Figure 4E), showing that profilin-MgATP-actin elongation is not coupled to Pi release. The coupling of profilin-actin elongation to ATP cleavage on the terminal subunit would result in the exposure of ADP-Pi actin subunits at the barbed end of growing filaments. At low actin concentrations, with an excess of profilin, the increase of 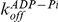 and 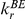 induced by profilin should then greatly lower the elongation rate and result in a higher fraction of ADP-actin subunits in the growing filament, i.e. a lower α*_ADP–Pi_*(*τ* = 0). Neither is observed: filaments grown with 0.25 µM actin and 2 µM profilin elongate with a rate 30% lower than their profilin-free counterparts, and their Pi decay profiles are identical (Figure 4E, inset). These results imply that filament growth from profilin-ATP-actin is not coupled to ATP cleavage on the terminal subunit.

## Discussion

### Elongation of Filaments from Profilin-Actin

Free energy balance calculations in the case of profilin-MgATP-actin polymerization indicate the involvement of ATP hydrolysis [18]. This idea is supported by the observation that profilin does not allow the elongation of filaments from MgADP-actin, CrATP-actin (which does not release its Pi), and CaATP-actin (which slowly hydrolyses ATP) [16],[31],[32]. A description of filament elongation from profilin-actin emerges from these results, where the dissociation of profilin from the barbed end following incorporation of one actin subunit in the filament is coupled to either ATP cleavage or Pi release and constitutes the rate-limiting step observed at high profilin-actin concentrations [31].

Kinosian et al. have challenged this view, by introducing the effect of profilin on the affinity of actin for MgATP in order to balance the energy diagram for profilin-actin elongation [15]. In another study, these authors conclude that profilin binds MgADP-G-actin with a high affinity (K_d_ = 0.4 µM) and that barbed end growth is supported by profilin-MgADP-G-actin [33]. This is contradicted by our results (Figure S10), which show that profilin has a weak affinity for MgADP-G-actin (K_d_ = 2.1 µM) and that profilin inhibits the elongation of filaments from MgADP-G-actin. We find identical concentrations of profilin-MgADP-G-actin at equilibrium with MgADP-F-actin and MgADP-G-actin when filaments are capped or non-capped, consistent with equilibrium thermodynamics of actin assembly in ADP.

Blanchoin and Pollard also argued against the coupling of ATP hydrolysis by rapidly and directly measuring the rate of ATP cleavage on filaments elongating in the presence or absence of profilin [14]. In addition, they find no higher limit for the elongation rate at high profilin-actin concentrations, indicating that the dissociation of profilin from the barbed end is extremely fast. However, technical difficulties in the normalization of the two kinetics have been discussed [16] and the issue of the coupling of profilin-actin elongation to ATP hydrolysis remains unsettled.

Our results show that the elongation of filaments from profilin-MgATP-actin is not coupled to Pi release in the filament. This indicates that the inability to elongate filaments from profilin-CrATP-actin [16] is not due to its very slow release of Pi, but may instead reflect a slow cleavage of CrATP (similar to CaATP-actin).

Our results also imply that the elongation of filaments from profilin-MgATP-actin is not coupled to ATP cleavage on the terminal subunit. This does not rule out coupling to ATP hydrolysis altogether, and our data can be interpreted with the hypothesis of either direct, indirect, or no coupling of ATP hydrolysis to elongation from profilin-MgATP-actin.

In the hypothesis of direct coupling, ATP cleavage could occur on the penultimate actin subunit as profilin dissociates from the barbed end, as proposed by Romero et al. [16] This model considers the two terminal subunits of the barbed end and is compatible with our data. The model of indirect coupling proposed by Yarmola and Bubb [18] is also compatible with our data. In fact, our result that profilin accelerates the dissociation of ADP-Pi-actin subunits from the barbed end (Figure 4C) validates one of the hypotheses underlying this model.

We show that profilin-MgADP-actin dissociates faster than MgADP-actin from barbed ends and does not productively associate with barbed ends. In contrast, the elongation of filaments from profilin-MgATP-actin occurs with a critical concentration and an association rate constant similar to that of actin, and is unaffected by free profilin up to 100 µM [14],[31]. Further work, beyond the scope of the present study, will have to be undertaken to fully understand the consequences of our new results in the biological function of profilin.

Since we find that profilin enhances the depolymerization of ADP-Pi- and ADP-actin, we propose that it should reinforce actin turnover in cells and enhance length fluctuations near the critical concentration, hereby facilitating their experimental observation. Based on our results, we expect that filament length fluctuations near the critical concentration should correspond to what can be computed for a random ATP hydrolysis, and not a vectorial one [10],[13].

### Experimental Outlook

Our observations allow us to determine the ADP/ADP-Pi-actin composition of actin filaments. Our setup could also be used to get information on the ATP-actin cap of growing actin filaments: in the case of a vectorial ATP cleavage mechanism, rapidly elongating filaments would grow long ATP caps that could last long enough to be observed by depolymerization. The elongation rates that can be reached using our setup are limited by spontaneous nucleation (in the absence of profilin), and do not allow us to draw significant conclusions on the ATP cap. However, this limitation could be eliminated by triggering the polymerization of actin inside the flow cell. This could be achieved by implementing a “mixer” in the setup [34] in order to expose G-actin to KCl only seconds before reaching the spectrin-actin seeds.

The results in this article show that accurate biochemical information can be derived from the observation of individual actin filaments in a microflow. The experimental setup we present here turns a microscope into a powerful tool for biochemical studies, as it circumvents most of the drawbacks of standard single filament microscopy in comparison to solution studies. It is versatile, and additional features, such as specific protein localization on the surface by patterning techniques, could further extend its potency. It should prove instrumental in the analysis of the mechanisms of actin regulation involved in many cellular processes.

## Materials and Methods

### Proteins and Buffers

Actin was purified from rabbit muscle [35]. Recombinant Profilin I from mouse was expressed in E. Coli and purified as described elsewhere [36]. Actin was labeled with Alexa488 succimidyl ester. The labeled fraction was 12% for our experiments, but 7%–20% labeling was used for control experiments. Cr-ATP was made as described elsewhere [24]. ADP-actin was obtained from ATP-actin using hexokinase and glucose. Spectrin-actin seeds were purified from human erythrocytes.

Standard elongation and depolymerization of filaments was done in F-buffer (5 mM TRIS pH 7.8, 0.2 mM ATP, 0.1 mM CaCl_2_, 0.01% NaN_3_, 100 mM KCl, 1 mM MgCl_2_, 0.2 mM EGTA) supplemented with 10 mM DTT and 1 mM DABCO to limit photobleaching. ATP-free buffer was used for experiments with ADP-actin. CrATP-actin filaments were elongated at pH 7. Buffer containing 100 mM Pi was obtained by diluting a mixture of 61.5% KH_2_PO_4_/38.5% K_2_HPO_4_ in Hepes buffer at pH 7.

### Microfluidics Experiments

Flow cells were made with Poly Dimethyl Siloxane (PDMS) from Sylgard mounted on standard glass coverslips that were previously cleaned in NaOH 1 M. Molds made of SU-8 photoresist were built at the ESPCI clean room (Paris), with the assistance of Hélène Berthet. The microchambers used in this study were Y- or trident-shaped, having two or three entry channels, respectively. The microchannels were 42 µm high and 200–800 µm wide. After adsorption of spectrin-actin seeds, the surface of the coverslip was passivated with Bovine Serum Albumin (Sigma).

Flow rates were controlled and monitored using a MAESFLO system (Fluigent, Paris). For each channel, the flow rate could be modulated instantly throughout the experiment, between zero and a few tens of µL/min. For flow rates lower than 1 µL/min the filaments fluctuated thermally away from the surface and were difficult to image. Above 5 µL/min, the filaments aligned with the flow, and the amplitude of thermal fluctuations was reduced. Observations were carried out between 1 and 3 mm downstream of the entry channel junction.

All measurements were carried out at room temperature.

### Image Acquisition and Analysis

Observations were carried out on an inverted Olympus IX71 microscope, with a 60× objective (and an additional 1.6× magnification in some cases), using TIRF or epifluorescence. Images were acquired by a Cascade II camera (Photometrics). The experiment was controlled using Metamorph. The time interval between images was typically 4–20 s, but longer intervals (30–120 s) were also used in control experiments. Image stacks were analyzed using ImageJ, and contrast was enhanced using the KymoToolBox plugin (available from fabrice.cordelieres@curie.u-psud.fr). Filament lengths were extracted from the images using the snake fit program from the laboratory of D. Vavylonis [37].

The local depolymerization velocity was estimated by fitting linearly the length-versus-time plot around a given point, over a symmetrical window of 4–12 time intervals. We verified that, over these time ranges, increasing the window of the linear fit had no effect other than reducing the noise in the velocity profile.

By monitoring the elongation of filaments, we have verified that filaments grew with a constant polymerization velocity *v_pol_*, which was compatible with the on-rates determined in solution assays: k_on_ = 10 µM^−1^ s^−1^ for actin, and k_on_ = 7 µM^−1^ s^−1^ for profilin-actin. Fragmentation events were rare, and pauses during elongation or annealing events were not observed. In some experiments, the filaments were not monitored during elongation, and their growth rate was computed by measuring the length before and after elongation, and dividing the difference by the duration of elongation. In these experiments, we could verify that nearly all filaments exhibited the expected elongation rate. The few filaments that did not, presumably due to fragmentation, were discarded.

The age τ is the time elapsed since the assembly of a portion of filament. Upon depolymerization, it is determined by the following equation: τ = t+(L(t = 0)−L(t))/*v_pol_*, where L(t) is the length of the depolymerizing filament, at time t after the onset of depolymerization.

In our computations we considered that each actin subunit contributes to 2.7 nm of the filament length.

### Control Experiments

We have performed experiments with different exposure times (10–50 ms in TIRF, and 100–500 ms in epifluorescence) and with different time intervals between acquisitions (4–120 s) and no effect on the depolymerization curves could be detected. We have monitored the depolymerization of filaments labeled with various fractions of Alexa488-actin (7%–20%), and no difference was observed. The effect of photobleaching was estimated by repeatedly measuring the length of fluorescent segments embedded in non-labeled filaments (fabricated by sequentially polymerizing filaments with labeled and non-labeled actin) and was found negligible in our experiments.

We have verified that the flow of liquid had no impact on the dynamics of the filaments, by performing the following measurements. Filaments were elongated under various constant flow rates, ranging from a few tens of nL/min to a few tens of µL/min, and the resulting elongations were the same. Filaments were depolymerized under constant flow rates, ranging from two to a few tens of µL/min, and the resulting depolymerization curves were the same. Filaments were depolymerized with flow rates that varied over a period of 30 s (25 s at 200 nL/min, followed by 5 s at a few tens of µL/min, during which the image was acquired) and the resulting depolymerization curves were the same.

We verified that the vicinity of the coverslip surface had no impact on the dynamics of the filaments, by performing the following measurements. 6 µm-diameter polystyrene carboxylate beads were incubated with spectrin-actin seeds and anchored to the bottom of the flow cell. We monitored filaments growing from the spectrin-actin seeds that were at least 3 µm above the coverslip surface. These filaments aligned with the flow, which maintained them entirely in the focal plane. The depolymerization curves were the same as for filaments grown from seeds anchored directly on the glass coverslip.

As a control for experiments with 100 mM Pi, depolymerization of filaments was monitored at pH 7 (Hepes buffer) with K_2_SO_4_ to adjust the ionic strength. The observed depolymerization was the same as in standard F-buffer.

## Supporting Information

Figure S1 Comparison of depolymerization traces from different individual filaments, under different conditions. Filaments were depolymerized in F-buffer after elongation from (A) MgATP-actin or (B) MgADP-actin. Filaments were depolymerized (C) in the presence of 20 µM profilin after elongation from MgATP-actin, or (D) in the presence of 40 µM profilin after elongation from MgADP-actin. Some traces have been shifted vertically in order to ease their comparison. For each filament, the depolymerization trace terminates when the filament becomes too short to be reliably measured, when it fragments, or when it pauses. Pauses (indicated here by a dotted line) are discarded during data analysis.
(PDF)

Figure S2 Pauses occurring during depolymerization are unrelated to the acceleration of depolymerization, which reflects the ADP-Pi content of the filament. Left: Length versus time for a filament depolymerizing with 80 µM profilin. Depolymerization is interrupted by a pause between 100 and 150 s after the beginning of depolymerization. Right: 1/*v_depol_* versus the age of F-actin, for the same filament, excluding the pause (blue diamonds) and exponential fit (black line).
(PDF)

Figure S3 Depolymerization of an ADP-actin filament obtained by aging. A filament grown with 2 µM MgATP-actin is then left to age at constant length in the presence of 0.1 µM actin (steady-state concentration for the barbed end) for 6 min, before initiating depolymerization at time t = 0.
(PDF)

Figure S4 The length of the ADP-Pi F-actin at growing barbed ends depends on the age of filaments. Filaments growing in coherent fashion in the presence of 3 µM G-actin (50% pyrene-labeled) and the indicated amounts of spectrin-actin seeds (s.a.s.) were depolymerized by 6-fold dilution in F buffer in the presence of 5 µM Latrunculin A as soon as 20% of actin was assembled. Time courses of growth (left) and depolymerization (right), with the first 100 s of smoothed depolymerization curves presented on the inset. The lag time is visible, and it is longer for younger filaments.
(PDF)

Figure S5 Direct fit of depolymerization curve to experimental data. The theoretical curve, given by the differential equation 12 of the supporting text, is fitted to the depolymerization curves of six filaments from one experiment. These curves were slightly shifted in a vertical direction to have a common initial length. Inset: The theoretical curve is fitted to a single experimental curve.
(PDF)

Figure S6 Simulation of the length during polymerization and depolymerization. The rates are specified in the Text S1. Top: A cap of ATP-actin is present during polymerization, but not during depolymerization. Number of ATP-subunits (red), ADP-Pi-subunits (black), ADP-subunits (green), and overall number (blue). Bottom: Fluctuations indicated by 20 randomly chosen trajectories.
(PDF)

Figure S7Comparison of simulations with analytical results. We simulated the polymerization and depolymerization of 10,000 filaments with rates as specified in Text S1, i.e. including ATP cleavage. Average values (blue dots) ± standard deviations (blue dashed lines) are depicted. The continuous red line is the solution of equation 12 of Text S1 for the same parameters as used in the simulations. The fit is sufficient, since the deviation is much smaller than the optical resolution. The small error is mainly caused by neglecting the cleavage step. In a further improved approximation, we could consider ATP cleavage by an effective release rate which takes both cleavage and release into account. Thus we replace 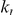 by *k_r_k_c_*/(*k_r_*+*k_c_*) in equation 12 of the Text S1. This yields the green line, which is in very good agreement with the simulations. Inset: The exponential relation between 
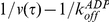
 and 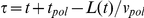 is also found for simulated trajectories. The agreement with the analytical results shows that fitting the experimental 1/*v*(*τ*) curves with an exponential indeed reveals the correct parameters.(PDF)

Figure S8 The depolymerization of ADP-actin filaments slows down upon exposure to Pi. A filament elongated from MgADP-actin was depolymerized in standard F-buffer for 60 s (blue diamonds) then in the presence of 25 mM Pi (red squares). Lines represent linear fits of the data. At this resolution, the transition to a slow depolymerization rate appears instantaneous upon exposure to Pi, as expected from the rapid Pi association to the barbed end reported by Fujiwara et al. [25]. In the presence of 25 mM Pi, ADP-actin filaments depolymerized at a rate of 0.64±16 subunits/s, which is also in agreement with the values reported in [25].
(PDF)

Figure S9 The effect of profilin during depolymerization is fully reversible. A depolymerizing filament is exposed to 100 µM profilin for 1 min and subsequently switched back to depolymerization in buffer without profilin.
(PDF)

Figure S10 Filaments do not elongate from MgADP-G-actin in the presence of profilin. (A) Barbed end and pointed end growths from 16.4 µM (blue) and 10.5 µM (red) MgADP-G-actin (2% pyrene-labeled) were initiated using 0.2 nM spectrin-actin seeds (open circles) or 10 nM gelsolin-actin seeds (closed squares), respectively, in the presence of the indicated amounts of profilin. The extent of F-actin assembled at equilibrium (reached in less than 1 h) was measured. Identical linear decrease in F-ADP-actin with capped and non-capped filaments shows that profilin binds MgADP-G-actin with K_d_ = 2.1 µM (this value was confirmed by measurements of tryptophan fluorescence quenching upon binding of profilin to actin—unpublished data) and that profilin-MgADP-G-actin does not productively associate with barbed nor pointed ends. Profilin-MgADP-G-actin hence accumulates in solution as described by [PA] = [P]_0_ A_c_/(A_c_+K_d_), where [P]_0_ is the total profilin concentration and A_c_ is the critical concentration for ADP-G-actin assembly at either barbed or pointed ends. In contrast, in Kinosian et al.'s view [33] the proposed productive association of profilin-MgADP-G-actin at barbed ends specifically would have led to a steeper decrease of F-actin concentration for capped filaments than for non-capped filaments in ADP, like the observed behavior in ATP. (B) Elongation rate of MgADP-actin filaments, in the presence of 5 µM MgADP-G-actin and the indicated amounts of profilin, measured on individual filaments in a microflow.
(PDF)

Text S1 (I) Additional information on the pauses that occur during depolymerization. (II) Theoretical analysis for the depolymerizaton of actin filaments, (III) analysis of length-versus-time data for depolymerizing filaments, (IV) stochastic simulations.
(PDF)

Video S1 Individual actin filament, elongated and subsequently depolymerized in a microfluidic flow. The filament is elongated for 6 min, from an initial length of 2.4 µm to a final length of 14.3 µm (30 s between images, acquired in TIRF microscopy). It is depolymerized by switching to buffer with no actin, and depolymerization lasts 12 min (20 s between images, acquired in epifluorescence microscopy). Liquid is flowing from left to right. Contrast was enhanced as described in the text.
(AVI)

## Abbreviations

F-actin: filamentous actin
G-actin: globular (monomeric) actin
Pi: inorganic Phosphate
TIRF: Total Internal Reflection Fluorescence

## References

[1] Le Clainche C, Carlier M. F (2008). Regulation of actin assembly associated with protrusion and adhesion in cell migration.. Physiol Rev.

[2] Pollard T. D, Borisy G. G (2003). Cellular motility driven by assembly and disassembly of actin filaments.. Cell.

[3] Bugyi B, Carlier M. F (2010). Control of actin filament treadmilling in cell motility.. Annu Rev Biophys.

[4] Carlier M. F, Pantaloni D (1986). Direct evidence for ADP-Pi-F-actin as the major intermediate in ATP-actin polymerization. Rate of dissociation of Pi from actin filaments.. Biochemistry.

[5] Carlier M. F, Pantaloni D (1988). Binding of phosphate to F-ADP-actin and role of F-ADP-Pi-actin in ATP-actin polymerization.. J Biol Chem.

[6] Isambert H, Venier P, Maggs A. C, Fattoum A, Kassab R (1995). Flexibility of actin filaments derived from thermal fluctuations. Effect of bound nucleotide, phalloidin, and muscle regulatory proteins.. J Biol Chem.

[7] Korn E. D, Carlier M. F, Pantaloni D (1987). Actin polymerization and ATP hydrolysis.. Science.

[8] Ohm T, Wegner A (1994). Mechanism of ATP hydrolysis by polymeric actin.. Biochim Biophys Acta.

[9] Carlier M. F, Pantaloni D, Korn E. D (1987). The mechanisms of ATP hydrolysis accompanying the polymerization of Mg-actin and Ca-actin.. J Biol Chem.

[10] Stukalin E. B, Kolomeisky A. B (2006). ATP hydrolysis stimulates large length fluctuations in single actin filaments.. Biophys J.

[11] Li X, Kierfeld J, Lipowsky R (2009). Actin polymerization and depolymerization coupled to cooperative hydrolysis.. Phys Rev Lett.

[12] Bindschadler M, Osborn EA, Dewey CF, McGrath JL (2004). A mechanistic model of the actin cycle.. Biophys J.

[13] Ranjith P, Mallick K, Joanny JF, Lacoste D (2010). Role of ATP-hydrolysis in the dynamics of a single actin filament.. Biophys J.

[14] Blanchoin L, Pollard T. D (2002). Hydrolysis of ATP by polymerized actin depends on the bound divalent cation but not profilin.. Biochemistry.

[15] Kinosian H. J, Selden L. A, Gershman L. C, Estes J. E (2004). Non-muscle actin filament elongation from complexes of profilin with nucleotide-free actin and divalent cation-free ATP-actin.. Biochemistry.

[16] Romero S, Didry D, Larquet E, Boisset N, Pantaloni D (2007). How ATP hydrolysis controls filament assembly from profilin-actin: implication for formin processivity.. J Biol Chem.

[17] Yarmola E. G, Bubb M. R (2006). Profilin: emerging concepts and lingering misconceptions.. Trends Biochem Sci.

[18] Yarmola E. G, Dranishnikov D. A, Bubb M. R (2008). Effect of profilin on actin critical concentration: a theoretical analysis.. Biophys J.

[19] Vavylonis D, Yang Q, O'Shaughnessy B (2005). Actin polymerization kinetics, cap structure, and fluctuations.. Proc Natl Acad Sci U S A.

[20] Fujiwara I, Takahashi S, Tadakuma H, Funatsu T, Ishiwata S (2002). Microscopic analysis of polymerization dynamics with individual actin filaments.. Nat Cell Biol.

[21] Kuhn J. R, Pollard T. D (2005). Real-time measurements of actin filament polymerization by total internal reflection fluorescence microscopy.. Biophys J.

[22] Brewer L. R, Bianco P. R (2008). Laminar flow cells for single-molecule studies of DNA-protein interactions.. Nat Methods.

[23] Kueh H. Y, Brieher W. M, Mitchison T. J (2008). Dynamic stabilization of actin filaments.. Proc Natl Acad Sci U S A.

[24] Valentin-Ranc C, Carlier M. F (1989). Evidence for the direct interaction between tightly bound divalent metal ion and ATP on actin. Binding of the lambda isomers of beta gamma-bidentate CrATP to actin.. J Biol Chem.

[25] Fujiwara I, Vavylonis D, Pollard T. D (2007). Polymerization kinetics of ADP- and ADP-Pi-actin determined by fluorescence microscopy.. Proc Natl Acad Sci U S A.

[26] Melki R, Fievez S, Carlier M. F (1996). Continuous monitoring of Pi release following nucleotide hydrolysis in actin or tubulin assembly using 2-amino-6-mercapto-7-methylpurine ribonucleoside and purine-nucleoside phosphorylase as an enzyme-linked assay.. Biochemistry.

[27] Blanchoin L, Pollard T. D (1999). Mechanism of interaction of Acanthamoeba actophorin (ADF/Cofilin) with actin filaments.. J Biol Chem.

[28] Combeau C, Carlier M. F (1988). Probing the mechanism of ATP hydrolysis on F-actin using vanadate and the structural analogs of phosphate BeF-3 and A1F-4.. J Biol Chem.

[29] Murakami K, Yasunaga T, Noguchi T. Q, Gomibuchi Y, Ngo K. X (2010). Structural basis for actin assembly, activation of ATP hydrolysis, and delayed phosphate release.. Cell.

[30] Bubb M. R, Yarmola E. G, Gibson B. G, Southwick F. S (2003). Depolymerization of actin filaments by profilin. Effects of profilin on capping protein function.. J Biol Chem.

[31] Gutsche-Perelroizen I, Lepault J, Ott A, Carlier M. F (1999). Filament assembly from profilin-actin.. J Biol Chem.

[32] Pantaloni D, Carlier M. F (1993). How profilin promotes actin filament assembly in the presence of thymosin beta 4.. Cell.

[33] Kinosian H. J, Selden L. A, Gershman L. C, Estes J. E (2002). Actin filament barbed end elongation with nonmuscle MgATP-actin and MgADP-actin in the presence of profilin.. Biochemistry.

[34] Tabeling P (2005). Introduction to microfluidics..

[35] Spudich J. A, Watt S (1971). The regulation of rabbit skeletal muscle contraction. I. Biochemical studies of the interaction of the tropomyosin-troponin complex with actin and the proteolytic fragments of myosin.. J Biol Chem.

[36] Gieselmann R, Kwiatkowski D. J, Janmey P. A, Witke W (1995). Distinct biochemical characteristics of the two human profilin isoforms.. Eur J Biochem.

[37] Smith M. B, Li H, Shen T, Huang X, Yusuf E (2010). Segmentation and tracking of cytoskeletal filaments using open active contours.. Cytoskeleton.

