# Supplementary figures and images for: Individual Actin Filaments in a Microfluidic Flow Reveal the Mechanism of ATP Hydrolysis and Give Insight Into the Properties of Profilin
Source: PLoS Biol. 2011 Sep 27;9(9):e1001161. doi: 10.1371/journal.pbio.1001161 (PMC3181223; doi:10.1371/journal.pbio.1001161)

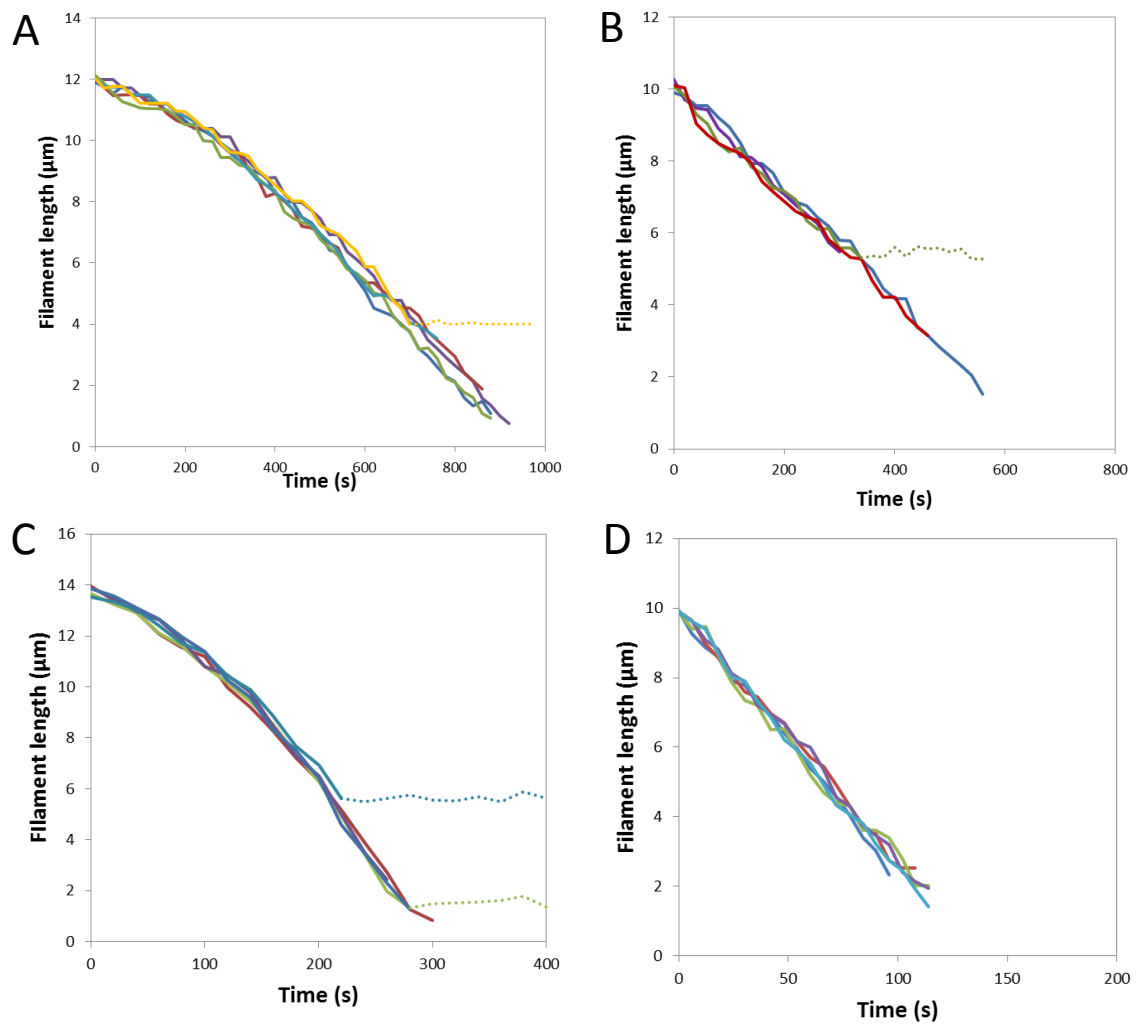

Jégou et al. Figure S1.

Supplement: Figure S1 — Comparison of depolymerization traces from different individual filaments, under different conditions. Filaments were depolymerized in F-buffer after elongation from (A) MgATP-actin or (B) MgADP-actin. Filaments were depolymerized (C) in the presence of 20 µM profilin after elongation from MgATP-actin, or (D) in the presence of 40 µM profilin after elongation from MgADP-actin. Some traces have been shifted vertically in order to ease their comparison. For each filament, the depolymerization trace terminates when the filament becomes too short to be reliably measured, when it fragments, or when it pauses. Pauses (indicated here by a dotted line) are discarded during data analysis. (PDF) [file pbio.1001161.s001.pdf]

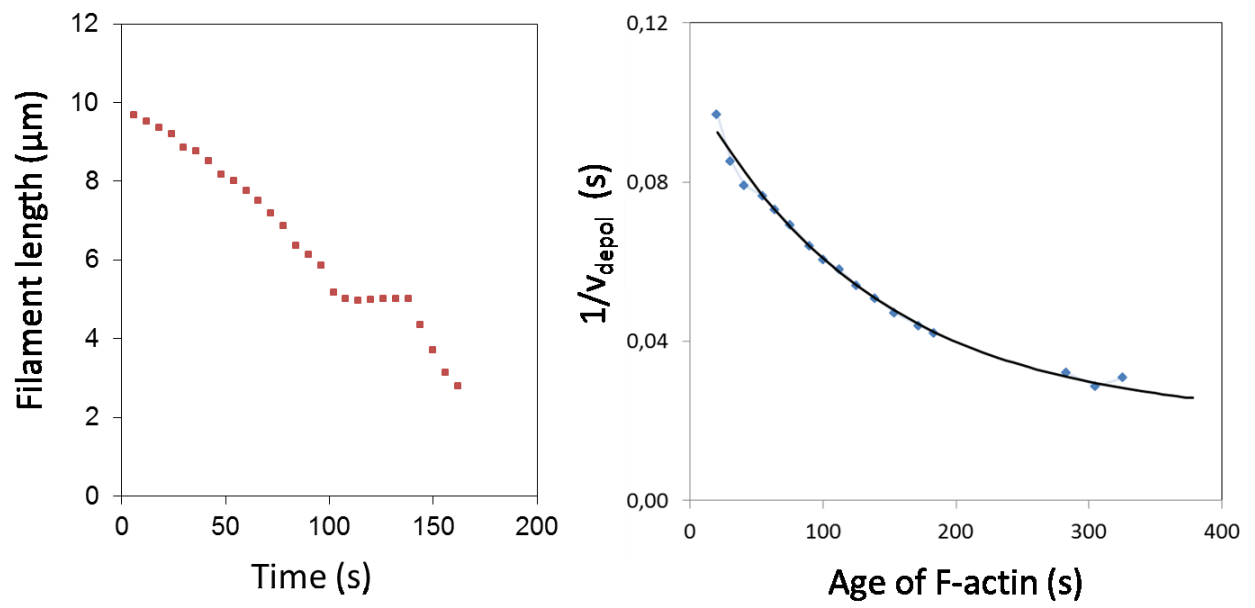

Jégou et al. Figure S2

Supplement: Figure S2 — Pauses occurring during depolymerization are unrelated to the acceleration of depolymerization, which reflects the ADP-Pi content of the filament. Left: Length versus time for a filament depolymerizing with 80 µM profilin. Depolymerization is interrupted by a pause between 100 and 150 s after the beginning of depolymerization. Right: 1/vdepol versus the age of F-actin, for the same filament, excluding the pause (blue diamonds) and exponential fit (black line). (PDF) [file pbio.1001161.s002.pdf]

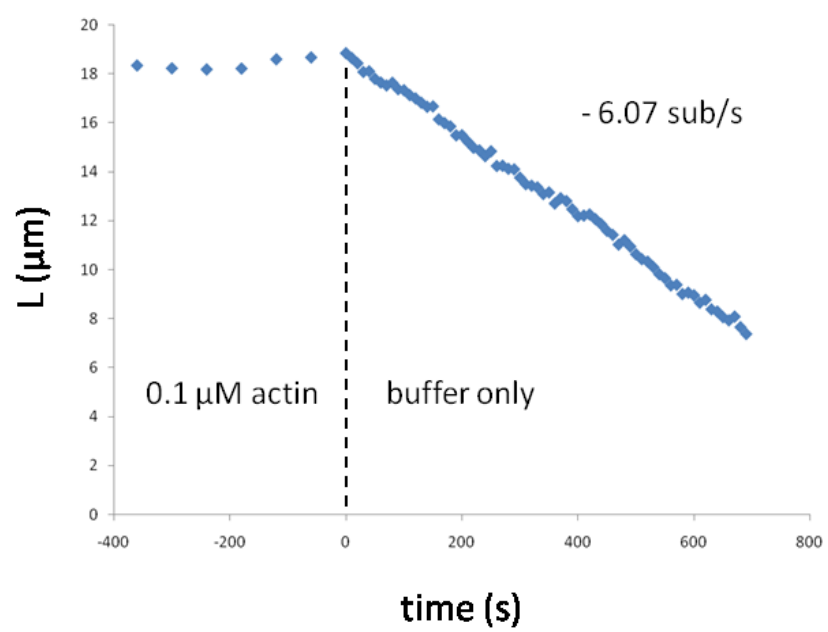

Jégou et al. Figure S3

Supplement: Figure S3 — Depolymerization of an ADP-actin filament obtained by aging. A filament grown with 2 µM MgATP-actin is then left to age at constant length in the presence of 0.1 µM actin (steady-state concentration for the barbed end) for 6 min, before initiating depolymerization at time t = 0. (PDF) [file pbio.1001161.s003.pdf]

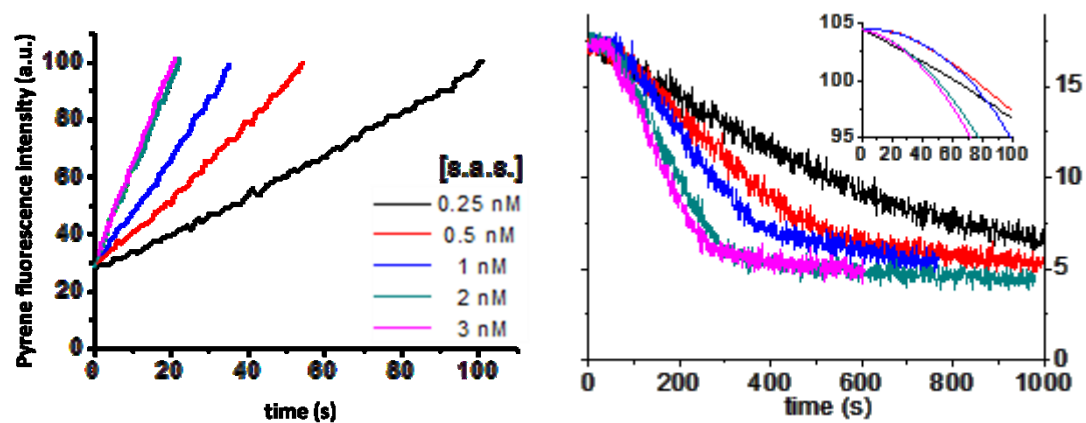

Jégou et al. Figure S4

Supplement: Figure S4 — The length of the ADP-Pi F-actin at growing barbed ends depends on the age of filaments. Filaments growing in coherent fashion in the presence of 3 µM G-actin (50% pyrene-labeled) and the indicated amounts of spectrin-actin seeds (s.a.s.) were depolymerized by 6-fold dilution in F buffer in the presence of 5 µM Latrunculin A as soon as 20% of actin was assembled. Time courses of growth (left) and depolymerization (right), with the first 100 s of smoothed depolymerization curves presented on the inset. The lag time is visible, and it is longer for younger filaments. (PDF) [file pbio.1001161.s004.pdf]

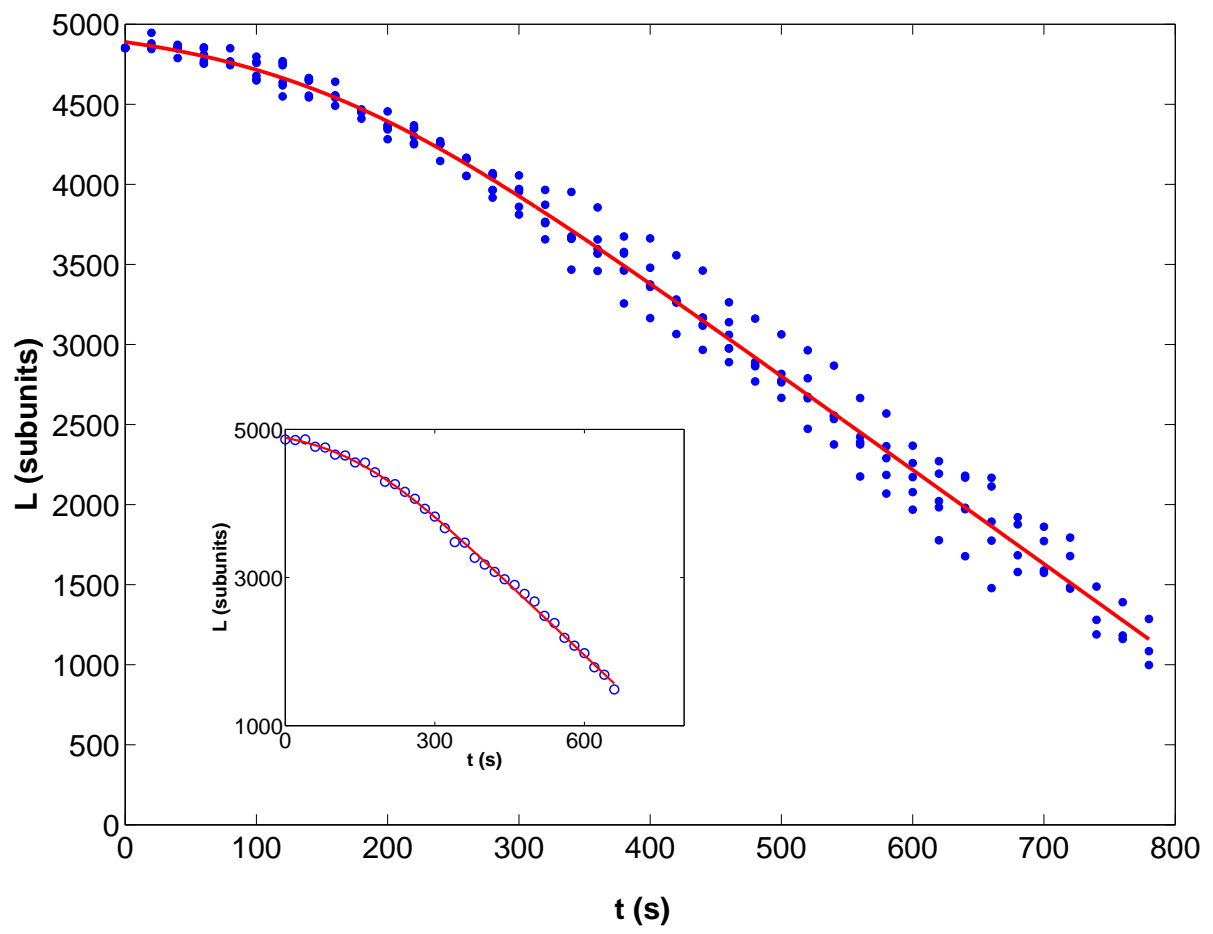

Jégou et al. Figure S5

Supplement: Figure S5 — Direct fit of depolymerization curve to experimental data. The theoretical curve, given by the differential equation 12 of the supporting text, is fitted to the depolymerization curves of six filaments from one experiment. These curves were slightly shifted in a vertical direction to have a common initial length. Inset: The theoretical curve is fitted to a single experimental curve. (PDF) [file pbio.1001161.s005.pdf]

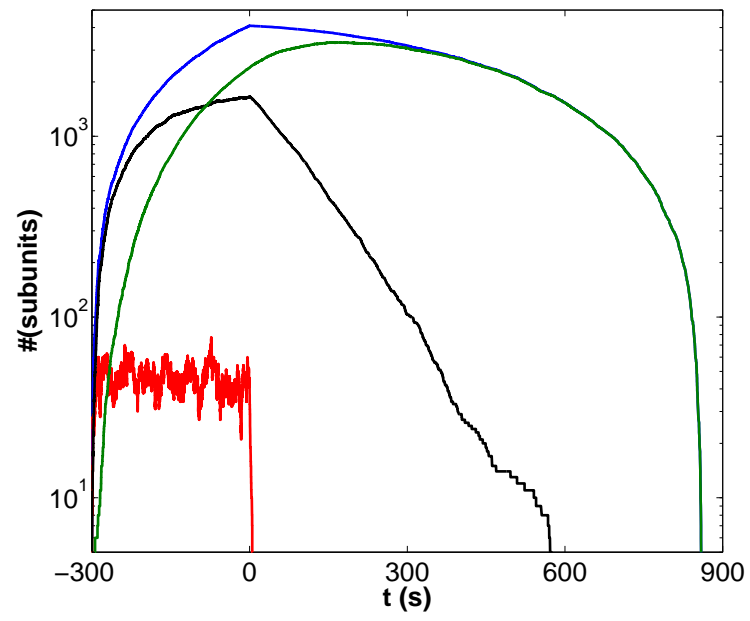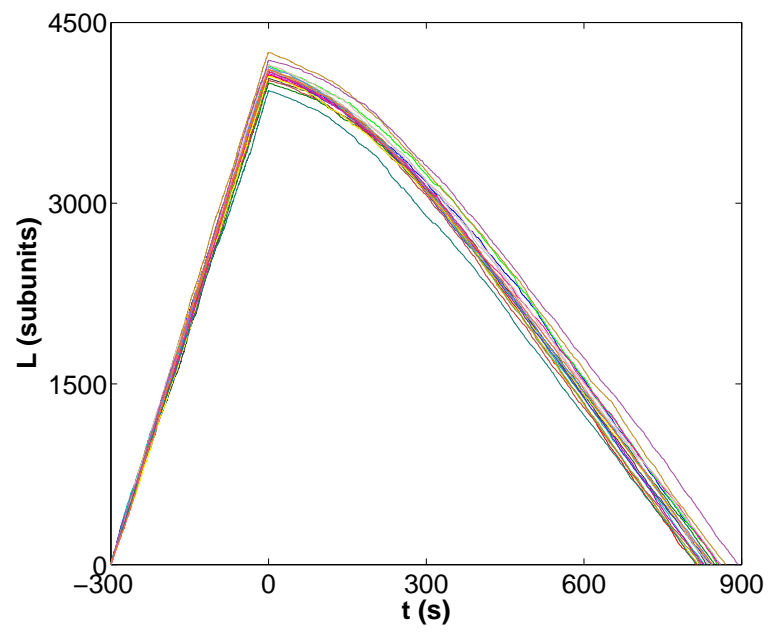

Jégou et al. Figure S6

Supplement: Figure S6 — Simulation of the length during polymerization and depolymerization. The rates are specified in the Text S1. Top: A cap of ATP-actin is present during polymerization, but not during depolymerization. Number of ATP-subunits (red), ADP-Pi-subunits (black), ADP-subunits (green), and overall number (blue). Bottom: Fluctuations indicated by 20 randomly chosen trajectories. (PDF) [file pbio.1001161.s006.pdf]

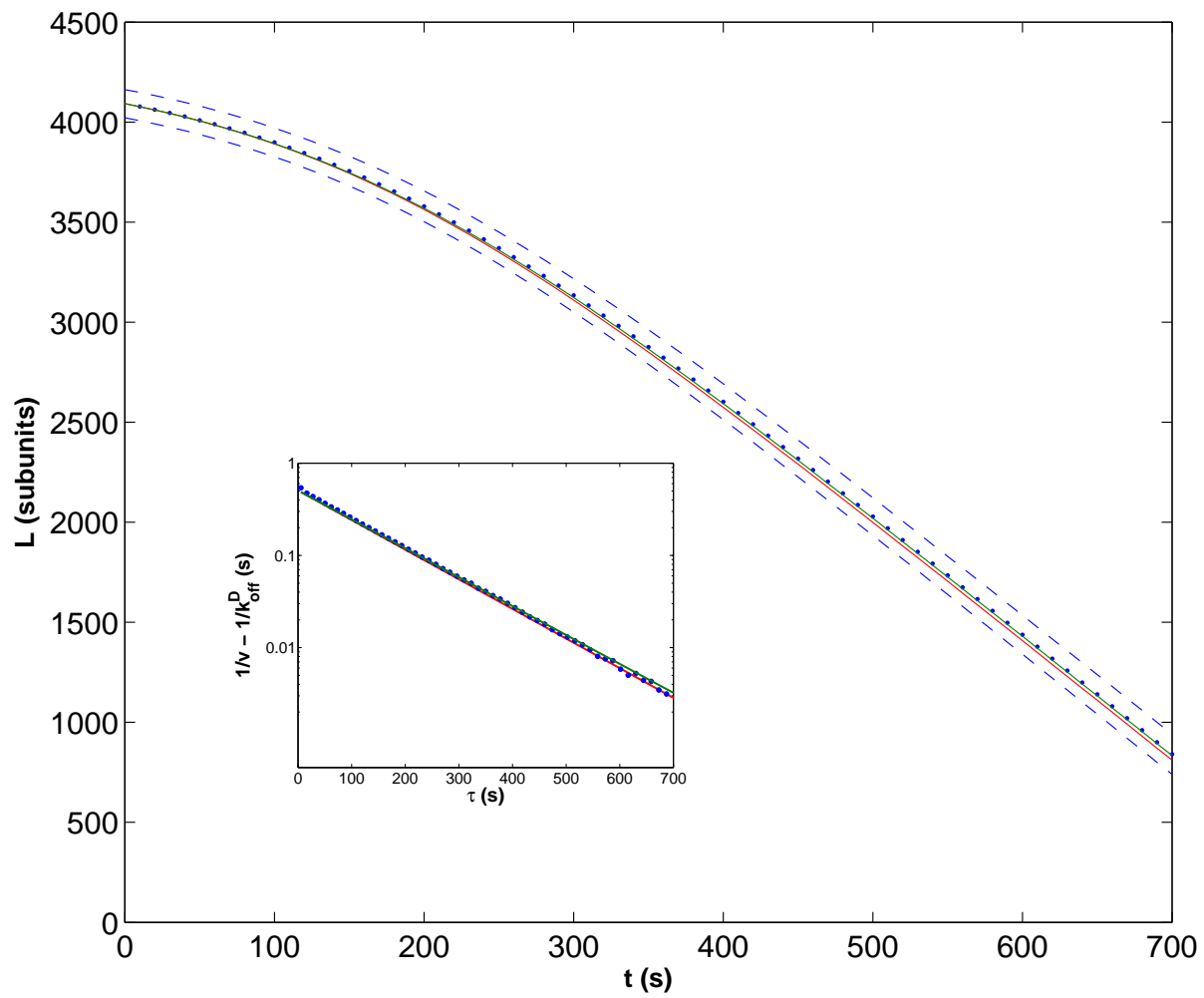

Jégou et al. Figure S7

Supplement: Figure S7 — Comparison of simulations with analytical results. We simulated the polymerization and depolymerization of 10,000 filaments with rates as specified in Text S1, i.e. including ATP cleavage. Average values (blue dots) ± standard deviations (blue dashed lines) are depicted. The continuous red line is the solution of equation 12 of Text S1 for the same parameters as used in the simulations. The fit is sufficient, since the deviation is much smaller than the optical resolution. The small error is mainly caused by neglecting the cleavage step. In a further improved approximation, we could consider ATP cleavage by an effective release rate which takes both cleavage and release into account. Thus we replace by krkc/(kr+kc) in equation 12 of the Text S1. This yields the green line, which is in very good agreement with the simulations. Inset: The exponential relation between and is also found for simulated trajectories. The agreement with the analytical results shows that fitting the experimental 1/v(τ) curves with an exponential indeed reveals the correct parameters. (PDF) [file pbio.1001161.s007.pdf]

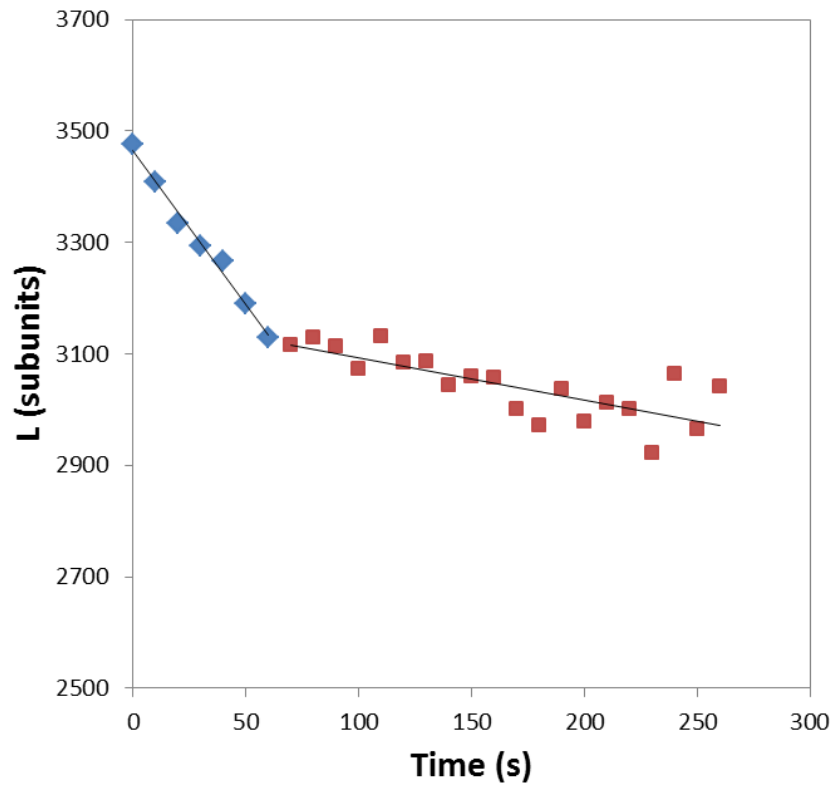

Jégou et al. Figure S8

Supplement: Figure S8 — The depolymerization of ADP-actin filaments slows down upon exposure to Pi. A filament elongated from MgADP-actin was depolymerized in standard F-buffer for 60 s (blue diamonds) then in the presence of 25 mM Pi (red squares). Lines represent linear fits of the data. At this resolution, the transition to a slow depolymerization rate appears instantaneous upon exposure to Pi, as expected from the rapid Pi association to the barbed end reported by Fujiwara et al. [25]. In the presence of 25 mM Pi, ADP-actin filaments depolymerized at a rate of 0.64±16 subunits/s, which is also in agreement with the values reported in [25]. (PDF) [file pbio.1001161.s008.pdf]

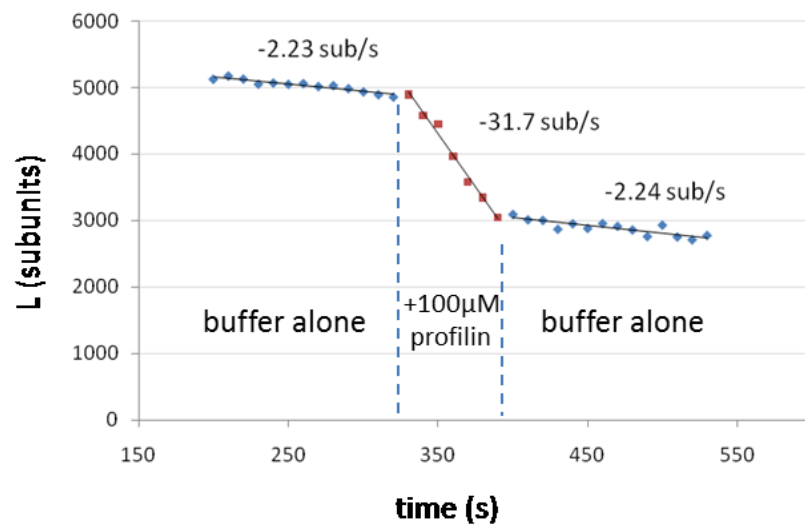

Jégou et al. Figure S9

Supplement: Figure S9 — The effect of profilin during depolymerization is fully reversible. A depolymerizing filament is exposed to 100 µM profilin for 1 min and subsequently switched back to depolymerization in buffer without profilin. (PDF) [file pbio.1001161.s009.pdf]

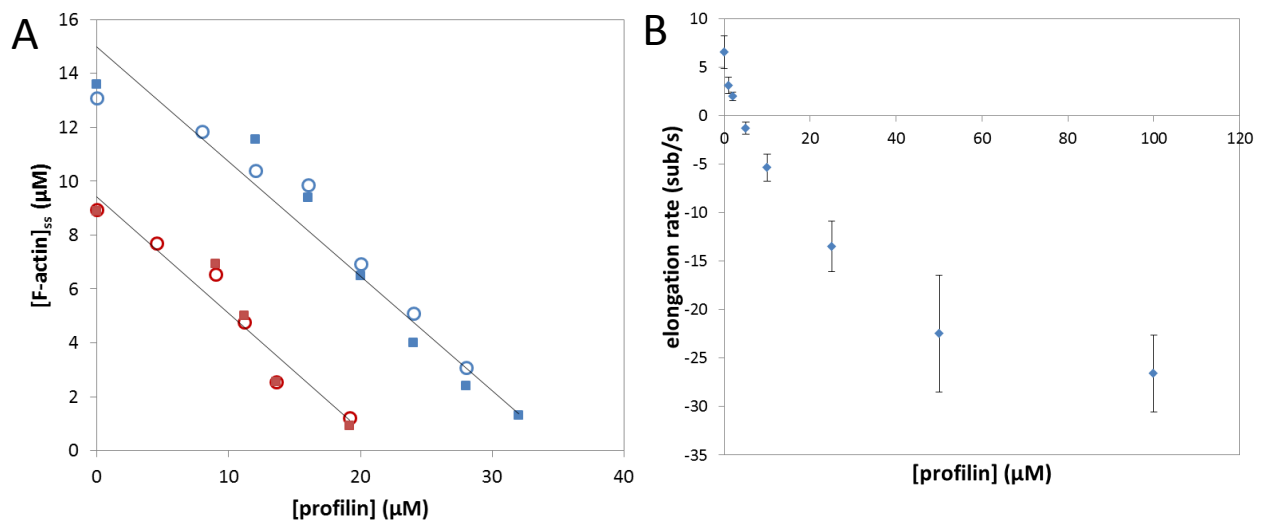

Jégou et al. Figure S10

Supplement: Figure S10 — Filaments do not elongate from MgADP-G-actin in the presence of profilin. (A) Barbed end and pointed end growths from 16.4 µM (blue) and 10.5 µM (red) MgADP-G-actin (2% pyrene-labeled) were initiated using 0.2 nM spectrin-actin seeds (open circles) or 10 nM gelsolin-actin seeds (closed squares), respectively, in the presence of the indicated amounts of profilin. The extent of F-actin assembled at equilibrium (reached in less than 1 h) was measured. Identical linear decrease in F-ADP-actin with capped and non-capped filaments shows that profilin binds MgADP-G-actin with Kd = 2.1 µM (this value was confirmed by measurements of tryptophan fluorescence quenching upon binding of profilin to actin—unpublished data) and that profilin-MgADP-G-actin does not productively associate with barbed nor pointed ends. Profilin-MgADP-G-actin hence accumulates in solution as described by [PA] = [P]0 Ac/(Ac+Kd), where [P]0 is the total profilin concentration and Ac is the critical concentration for ADP-G-actin assembly at either barbed or pointed ends. In contrast, in Kinosian et al.'s view [33] the proposed productive association of profilin-MgADP-G-actin at barbed ends specifically would have led to a steeper decrease of F-actin concentration for capped filaments than for non-capped filaments in ADP, like the observed behavior in ATP. (B) Elongation rate of MgADP-actin filaments, in the presence of 5 µM MgADP-G-actin and the indicated amounts of profilin, measured on individual filaments in a microflow. (PDF) [file pbio.1001161.s010.pdf]
