# Supplementary material for: Individual Actin Filaments in a Microfluidic Flow Reveal the Mechanism of ATP Hydrolysis and Give Insight Into the Properties of Profilin
Source: PLoS Biol. 2011 Sep 27;9(9):e1001161. doi: 10.1371/journal.pbio.1001161 (PMC3181223; doi:10.1371/journal.pbio.1001161)
Supplement: Text S1 — (I) Additional information on the pauses that occur during depolymerization. (II) Theoretical analysis for the depolymerizaton of actin filaments, (III) analysis of length-versus-time data for depolymerizing filaments, (IV) stochastic simulations. (PDF) [file pbio.1001161.s011.pdf]

Supporting Text - Jégou et al.

I. PAUSES DURING DEPOLYMERIZATION

Pauses were observed during the depolymerization of individual filaments grown from ADP-, CrATP- or MgATP-actin, in the presence or in the absence of profilin. As discussed in the main text, these pauses did not affect the depolymerization traces up to the interruption. Moreover, as can be seen from Fig. S1 and Fig. S2, the occurrence of a pause did not affect the depolymerization velocity for segments depolymerizing before or after the pause. The mechanism underlying the pausing events will be elucidated elsewhere [T. Niedermayer, A. Jégou, E. Helfer, G. Romet-Lemonne, M.-F. Carlier, R. Lipowsky, *in preparation*].

II. THEORETICAL ANALYSIS

We analyze the filament disassembly which is controlled by the dynamics of the nucleotide state of the actin subunit at the barbed end. As discussed in the main text, ATP is quickly cleaved, so we may only consider ADP-Pi-actin and ADP-actin subunits, respectively. This simplification is validated by the simulations described in section IV.

A. Random phosphate release

We consider the average depolymerization velocity of an ensemble of N filaments

$$v_{\rm depol}(t) \equiv \frac{1}{N}\sum_{i=1}^{N} v^{i}_{\rm depol}(t), \tag{1}$$

where the individual depolymerization velocities $v^{i}_{\rm depol}(t)$ are solely determined by the state of the subunit at the barbed end of the i-th filament. As discussed in the main text, an ADP-Pi-subunit can either dissociate directly with the rate $k^{\rm ADP\text{-}Pi}_{\rm off}$, or first release its phosphate with the rate $k^{\rm BE}_{\rm r}$ and then dissociate as an ADP-subunit with the rate $k^{\rm ADP}_{\rm off}$, see fig. 3 in the main text. In our experiments, we do not distinguish between these two routes. Thus we define an effective ADP-Pi-actin depolymerization rate $v^{\rm ADP\text{-}Pi}_{\rm depol}$ as the inverse of the mean time that it takes for an ADP-Pi-subunit to depart from the barbed end. It can be computed considering the following. The fraction $k^{\rm ADP\text{-}Pi}_{\rm off}/(k^{\rm BE}_{\rm r} + k^{\rm ADP\text{-}Pi}_{\rm off})$ of the ADP-Pi-subunits at the barbed end dissociate directly, while the fraction $k^{\rm BE}_{\rm r}/(k^{\rm BE}_{\rm r} + k^{\rm ADP\text{-}Pi}_{\rm off})$ first releases its phosphate before dissociating as ADP-actin. The dwell time of the initial ADP-Pi-state is given by $1/(k^{\rm BE}_{\rm r} + k^{\rm ADP\text{-}Pi}_{\rm off})$, and the dwell time of the ADP-state is given by $1/k^{\rm ADP}_{\rm off}$. In consequence, the

combination of the two routes leads to

$$v_{\rm depol}^{\rm ADP\text{-}Pi} = \frac{1}{\frac{k_{\rm off}^{\rm ADP\text{-}Pi}}{k_{\rm r}^{\rm BE}+k_{\rm off}^{\rm ADP\text{-}Pi}} \cdot \frac{1}{k_{\rm r}^{\rm BE}+k_{\rm off}^{\rm ADP\text{-}Pi}} + \frac{k_{\rm r}^{\rm BE}}{k_{\rm r}^{\rm BE}+k_{\rm off}^{\rm ADP\text{-}Pi}} \cdot \left(\frac{1}{k_{\rm r}^{\rm BE}+k_{\rm off}^{\rm ADP\text{-}Pi}} + \frac{1}{k_{\rm off}^{\rm ADP}}\right)} = \frac{(k_{\rm off}^{\rm ADP\text{-}Pi} + k_{\rm r}^{\rm BE})k_{\rm off}^{\rm ADP}}{k_{\rm off}^{\rm ADP} + k_{\rm r}^{\rm BE}}. \tag{2}$$

ADP-subunits that reach the barbed end dissociate directly with the rate $k_{\rm off}^{\rm ADP}$, giving rise to a depolymerization velocity $v_{\rm depol}^{\rm ADP}$. Consequently, the average depolymerization velocity is given by

$$v_{\rm depol}(t) = \mathcal{P}_1(t) v_{\rm depol}^{\rm ADP\text{-}Pi} + (1 - \mathcal{P}_1(t)) v_{\rm depol}^{\rm ADP}, \tag{3}$$

where $\mathcal{P}_1(t)$ and $(1 - \mathcal{P}_1(t))$ are the probabilities that the terminal subunit is in the ADP-Pi- and ADP-state, respectively. As phosphate release on the barbed end is not considered explicitly, but via the effective depolymerization velocity $v_{\rm depol}^{\rm ADP\text{-}Pi}$, the time evolution of the probability $\mathcal{P}_1(t)$ is governed by

$$\frac{\partial \mathcal{P}_1(t)}{\partial t} = -v_{\rm depol}^{\rm ADP\text{-}Pi} \mathcal{P}_1(t)(1 - \mathcal{P}_2(t)) + v_{\rm depol}^{\rm ADP}(1 - \mathcal{P}_1(t))\mathcal{P}_2(t), \tag{4}$$

where $\mathcal{P}_2(t)$ is the probability that the penultimate subunit is in the ADP-Pi-state. The first term on the r.h.s. of the equation accounts for the depolymerization of an ADP-Pi-subunit (both direct dissociation and phosphate release followed by dissociation of an ADP-subunit, see equation 2) and an ADP-subunit on the penultimate position. The second term accounts for the opposite situation: Dissociation of an ADP-subunit followed by an ADP-Pi-subunit.

During fast elongation, a cap of ATP-actin is present at the growing barbed end. This cap prevents the exposure of ADP-Pi-subunits to the barbed end which would result in enhanced phosphate release, cf. section IV. Thus the probability for the penultimate subunit to be in the ADP-Pi-state decays exponentially with its age τ, i.e. the time since it has been incorporated into the filament:

$$\mathcal{P}_2(t) = e^{-k_{\rm r}\tau}, \tag{5}$$

Filaments elongated with a constant velocity $v_{\rm pol}$ during the polymerization phase. Thus the age of a subunit is a linear function of its position within the filament. Moreover, during depolymerization, the penultimate subunit comoves with the tip of the filament. Thus the age of the penultimate subunit is a function of both the filament length $L(t)$ and the time t since the initiation of depolymerization, cf. fig. 1g in the main text:

$$\tau(t, L) = t + \tau_{\rm pol} - L(t)/v_{\rm pol}, \tag{6}$$

where τ_{pol} is the known duration of polymerization. In consequence, the differential equation

$$\frac{\partial \mathcal{P}_1(t)}{\partial t} = -v_{\text{depol}}^{\text{ADP-Pi}} \mathcal{P}_1(t) - (v_{\text{depol}}^{\text{ADP}} - v_{\text{depol}}^{\text{ADP-Pi}}) \mathcal{P}_1(t) e^{-k_{\text{r}}\tau(t,L)} + v_{\text{depol}}^{\text{ADP}} e^{-k_{\text{r}}\tau(t,L)}, \tag{7}$$

with the initial condition

$$\mathcal{P}_1(0) = 1, \tag{8}$$

determines $\mathcal{P}_1(t)$ and via equation 3 the depolymerization velocity. This leads to a second order differential equation for $L(t)$ which describes the depolymerization dynamics. Since phosphate release within the filament is much slower than dissociation, the asymptotic approximation

$$\frac{\partial \mathcal{P}_1(t)}{\partial t} / v_{\text{depol}}^{\text{ADP-Pi}} \approx 0 \tag{9}$$

can be employed, leading to

$$\mathcal{P}_1(\tau) \approx \frac{v_{\text{depol}}^{\text{ADP}} e^{-k_{\text{r}}\tau}}{(v_{\text{depol}}^{\text{ADP}} - v_{\text{depol}}^{\text{ADP-Pi}}) e^{-k_{\text{r}}\tau} + v_{\text{depol}}^{\text{ADP-Pi}}}. \tag{10}$$

With eq. 3, we derive equation (1) of the main text as an asymptotic approximation for the depolymerization velocity in the case of random phosphate release:

$$\frac{1}{v_{\text{depol}}(\tau)} \approx \frac{1}{v_{\text{depol}}^{\text{ADP}}} + \left(\frac{1}{v_{\text{depol}}^{\text{ADP-Pi}}} - \frac{1}{v_{\text{depol}}^{\text{ADP}}} \right) e^{-k_{\text{r}}\tau}. \tag{11}$$

The experimental data are given as length vs time curves. Thus, for the purpose of data analysis, it is most suitable to reformulate this relation for $v(\tau)$ as a differential equation for $L(t)$:

$$\frac{\partial L(t)}{\partial t} \approx \frac{-1}{\frac{1}{v_{\text{depol}}^{\text{ADP}}} + \left(\frac{1}{v_{\text{depol}}^{\text{ADP-Pi}}} - \frac{1}{v_{\text{depol}}^{\text{ADP}}} \right) e^{-k_{\text{r}}\tau(t,L)}}. \tag{12}$$

Partial derivatives with respect to the parameters can be calculated in order to fit the resulting curve to the experimental data, cf. section III.

B. Vectorial phosphate release

In the vectorial model, the phosphate can only be released from a ADP-Pi-subunit adjacent to an ADP-subunit. Thus there is an interface between a segment of ADP-subunits at the pointed end side and another segment of ADP-Pi-subunits at the barbed end side, cf. fig. 1a of the main text. This interface moves with a velocity given by

the release rate k_r towards the barbed end. Meanwhile, the barbed end moves with the depolymerization velocity of ADP-Pi-subunits $v_{\text{depol}}^{\text{ADP-Pi}}$ in the opposite direction. When the interface reaches the barbed end, it vanishes and the filament continues to shrink with the depolymerization velocity $v_{\text{depol}}^{\text{ADP}}$ of ADP-subunits. Thus the function $L(t)$ exhibits a kink when the interface reaches the end at time τ_1:

$$L(t) = \begin{cases} L_0 - v_{\text{depol}}^{\text{ADP-Pi}} t & \text{for} \quad t \leq \tau_1 \\ L_0 + (v_{\text{depol}}^{\text{ADP}} - v_{\text{depol}}^{\text{ADP-Pi}})\tau_1 - v_{\text{depol}}^{\text{ADP}} t & \text{for} \quad t > \tau_1. \end{cases} \tag{13}$$

Note that the ensemble average exhibits a smooth transition from slope $v_{\text{depol}}^{\text{ADP-Pi}}$ to $v_{\text{depol}}^{\text{ADP}}$ as the period τ_1 can slightly vary within the filament ensemble. However, the distribution of τ_1 within the filament population is very narrow. It will become clear in the next section that the vectorial model does not fit the data.

To be experimentally detectable, the rate of phosphate release of the ADP-Pi-subunit at the interface must be of the same order as the dissociation rate. However, equation (1) in the main text still holds, since there is no change in the ADP-Pi content within the distinct segments.

III. DATA ANALYSIS OF FILAMENT LENGTHS

A. Vectorial vs random mechanism

In this section, we formally check that the depolymerization velocity is continuously increasing from $v_{\text{depol}}^{\text{ADP-Pi}}$ to $v_{\text{depol}}^{\text{ADP}}$, consistent with the random mechanism of phosphate release, rather than suddenly changing from $v_{\text{depol}}^{\text{ADP-Pi}}$ to $v_{\text{depol}}^{\text{ADP}}$, as predicted by the vectorial mechanism. We do this without transforming the original $L(t)$ into $v(\tau)$ data. Thus we fit both a piecewise-linear function as prescribed by the vectorial model and $L(t)$ as given by equation 12 of the random model to the experimental data. The sum of squared residuals SSR provides a relative measure to compare the quality of both fits. With an average of $SSR_{\text{random}} = 9.3 \times 10^4$, compared to $SSR_{\text{vectorial}} = 1.25 \times 10^5$, we find that the model of random phosphate release describes the data better, consistent with the continuous course of $v(\tau)$.

It follows from a theoretical analysis of the vectorial model that the times τ_1 when the putative interface between ADP-Pi- and ADP-subunits reaches the barbed end are almost identical within the filament populations. However, fitting the data from each filament with a piecewise linear function leads to a broad τ_1 distribution, indicating another inconsistency of the data with the vectorial model.

B. Numerical values of kinetic parameters

In this section, we fit the $L(t)$ curve of the random model, given by equation 12, to the experimental data. In the fitting procedure, the known values of τ_{pol} and v_{pol} are fixed to determine the three unknown parameters $v_{\text{depol}}^{\text{ADP-Pi}}$,

$v_{\rm depol}^{\rm ADP}$, and k_r. This fitting approach is intended as a comparison for the method described in the main text, i.e. conversion of $L(t)$ curves into $1/v(\tau)$ curves and fitting them with an exponential.

We proceed in two different ways. First, we fit individual curves to each of the $N = 20$ filaments, cf. inset of Fig. S5, to obtain a set of $N = 20$ numerical values for each parameter. The means and standard deviations are given by $v_{\rm depol}^{\rm ADP} = (6.3 \pm 1.5)$ subunits/s, $v_{\rm depol}^{\text{ADP-Pi}} = (1.7 \pm 0.7)$ subunits/s and $k_r = (0.0083 \pm 0.0043)$/s. These results are in agreement with the values determined in the main text.

The other option for an analysis is to fit a single curve simultaneously to the data from all filaments. This gives the best estimate for the parameters. However, the $N = 20$ data sets were not obtained from a single, but 5 individual experiments whose $\tau_{\rm pol}$ and $v_{\rm pol}$ differ. Thus only the filament data within one experiment can be fitted simultaneously, cf. Fig. S5.

We obtain the following values for the weighted total averages of the parameters: $v_{\rm depol}^{\rm ADP} = 6.0$ subunits/s, $v_{\rm depol}^{\text{ADP-Pi}} =$ 1.5 subunits/s and $k_r = 0.0074$/s, in agreement with both the values determined before and the values determined with the method described in the main text. Equation 2 can be restated as

$$k_{\rm r}^{\rm BE} = \frac{(v_{\rm depol}^{\text{ADP-Pi}} - k_{\rm off}^{\text{ADP-Pi}})k_{\rm off}^{\rm ADP}}{k_{\rm off}^{\rm ADP} - v_{\rm depol}^{\text{ADP-Pi}}}, \tag{14}$$

and with $k_{\rm off}^{\text{ADP-Pi}} = 0.16$/s and $k_{\rm off}^{\rm ADP} = 5.8$/s, as given in the main text, we obtain $k_{\rm r}^{\rm BE} = 1.8$.

IV. SIMULATIONS

In the analytical calculations for the random release mechanism which finally led to equation 12, or equivalently equation (1) in the main text, the following simplifications were made .

- It was assumed that ATP-actin has a infinitely short lifetime within the filament. This is justified by the largeness of the known cleavage rate $k_{\rm c} = 0.3$/s [ref 17 in the main text] compared to the release rate $k_{\rm r}$.

- Phosphate release at the barbed end does not take place during polymerization. Even though we neglect ATP-actin-subunits in terms of hydrolysis, we assume that a cap of ATP-actin is present during polymerization which prevents ADP-Pi-subunits from loosing their phosphate by being exposed at the barbed end. This assumption is justified by the smallness of the cleavage rate $k_{\rm c} = 0.3$/s compared to the association rates in the experiment which are typically larger than 10/s.

- When considering the departure of an ADP-Pi-subunit, we do not distinguish between the two possible routes (cf. fig. 3 of the main text), but use an effective depolymerization rate $v_{\rm depol}^{\text{ADP-Pi}}$ given by 2. However this can only lead to errors on the scale of single subunits.

- As discussed in the main text, the phosphate release rate $k_{\rm r}$ is much smaller than the dissociation rates $k_{\rm off}^{\text{ADP-Pi}}$ and $k_{\rm off}^{\rm ADP}$. Thus we assume that $\mathcal{P}_1(t)$ is constant on depolymerization time scales.

In order to validate these simplifications, we use the Gillespie algorithm [1] which gives exact stochastic trajectories, to simulate the polymerization and depolymerization of filaments.

The following stochastic processes, which are known to play a role in actin dynamics, are taken into account. ATP cleavage with the rate $k_\mathrm{c} = 0.3/\mathrm{s}$ [ref. 17 in the main text]; phosphate release with the rate $k_\mathrm{r} = 0.0074/\mathrm{s}$; enhanced phosphate release at the barbed end with the rate $k_\mathrm{r}^{\mathrm{BE}} = 1.8/\mathrm{s}$; association of ATP-actin with the rate $k_{\mathrm{on}}^{\mathrm{ATP}} = 15/\mathrm{s}$ (only during polymerization phase which lasted for $\tau_{\mathrm{pol}} = 300/\mathrm{s}$); dissociation of ATP-actin with the rate $k_{\mathrm{off}}^{\mathrm{ATP}} = 1.4/\mathrm{s}$ [ref. 21 in the main text]; dissociation of ADP-Pi-actin with the rate $k_{\mathrm{off}}^{\mathrm{ADP\text{-}Pi}} = 0.16/\mathrm{s}$; and dissociation of ADP-actin with the rate $k_{\mathrm{off}}^{\mathrm{ADP}} = 6/\mathrm{s}$. Apart from k_c and $k_{\mathrm{off}}^{\mathrm{ATP}}$, the numerical values for the rates were taken from the last section.

It is shown in Fig. S7, that the simulations indeed justify the simplifications which were made to obtain the analytical results, and especially equation (1) in the main text. The irrelevance of the ATP-subunits as an intermediate during depolymerization and its importance during polymerization as a protective cap against enhanced phosphate release becomes clear with Fig. S6. The simulations also indicate the length fluctuations which follow from the stochasticity of the involved transitions. During the first instances of growth, the polymerization trajectories tend to diverge, leading to a spreading in the length at the beginning of depolymerization. During the depolymerization process, this spread varies only slightly.

---

[1] D. T. Gillespie, Journal of Physical Chemistry **81**, 2340 (1977).
